# Supplementary material for: Preoperative Serum Calcitonin and Its Correlation with Extent of Lymph Node Metastasis in Medullary Thyroid Carcinoma
Source: Cancers (Basel). 2020 Oct 9;12(10):2894. doi: 10.3390/cancers12102894 (PMC7601718; doi:10.3390/cancers12102894)
Supplement: Supplementary file 1 [file cancers-12-02894-s001.pdf]

*Supplementary Materials*

# Preoperative Serum Calcitonin and Its Correlation with Extent of Lymph Node Metastasis in Medullary Thyroid Carcinoma

**Hyunju Park, Jun Park, Min Sun Choi, Jinyoung Kim, Hosu Kim, Jung Hee Shin, Jung-Han Kim, Jee Soo Kim, Sun Wook Kim, Jae Hoon Chung and Tae Hyuk Kim**

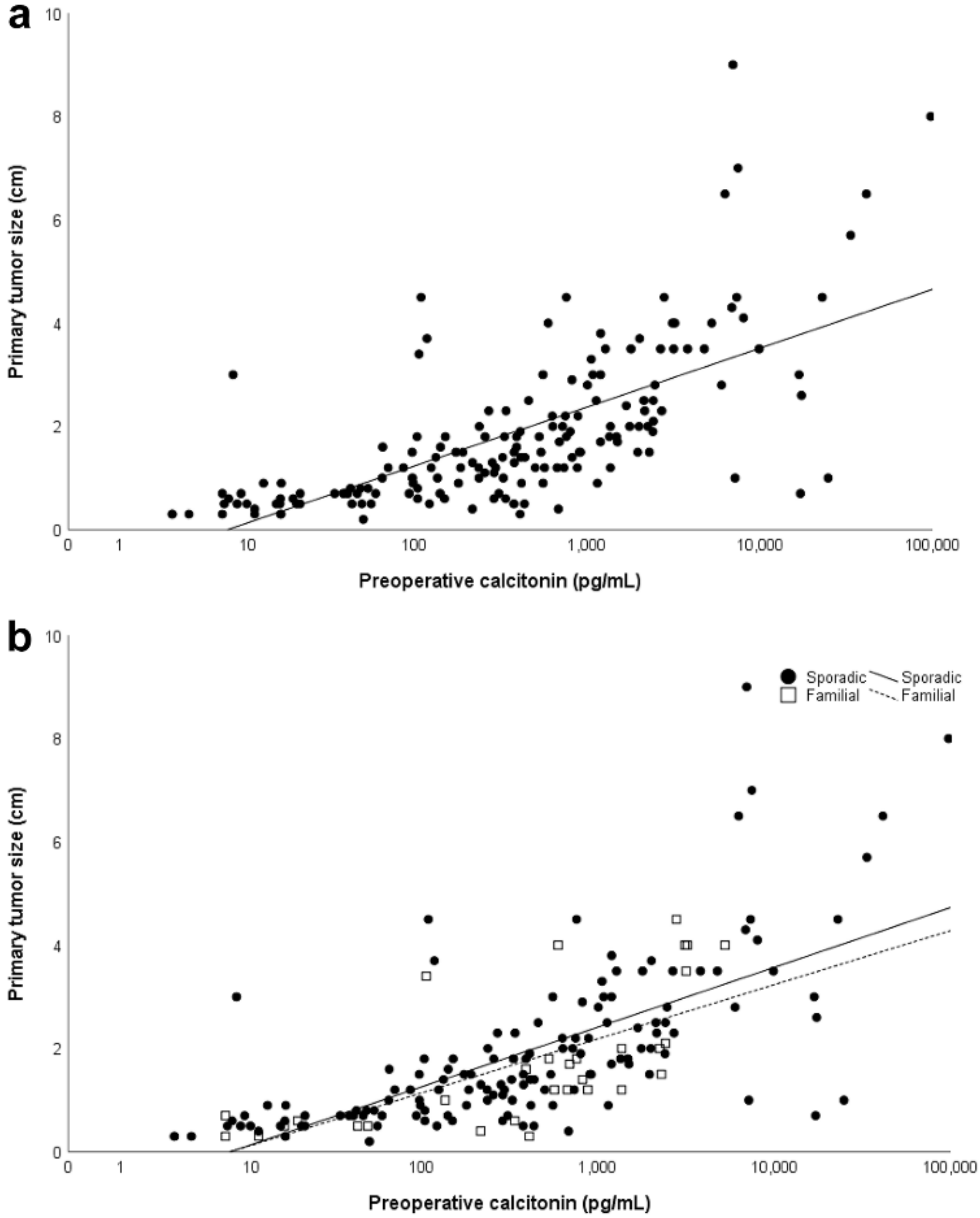


**Figure S1.** Correlation between log-transformed preoperative calcitonin level and primary tumour size. (A) All patients regardless of tumour type ($R^2 = 0.746$ and $p < 0.001$). (B) Patients divided into sporadic and hereditary medullary carcinoma groups ($R^2 = 0.464$ and $p < 0.01$ for sporadic medullary thyroid carcinoma; $R^2 = 0.444$ and $p < 0.001$ for hereditary medullary thyroid carcinoma).

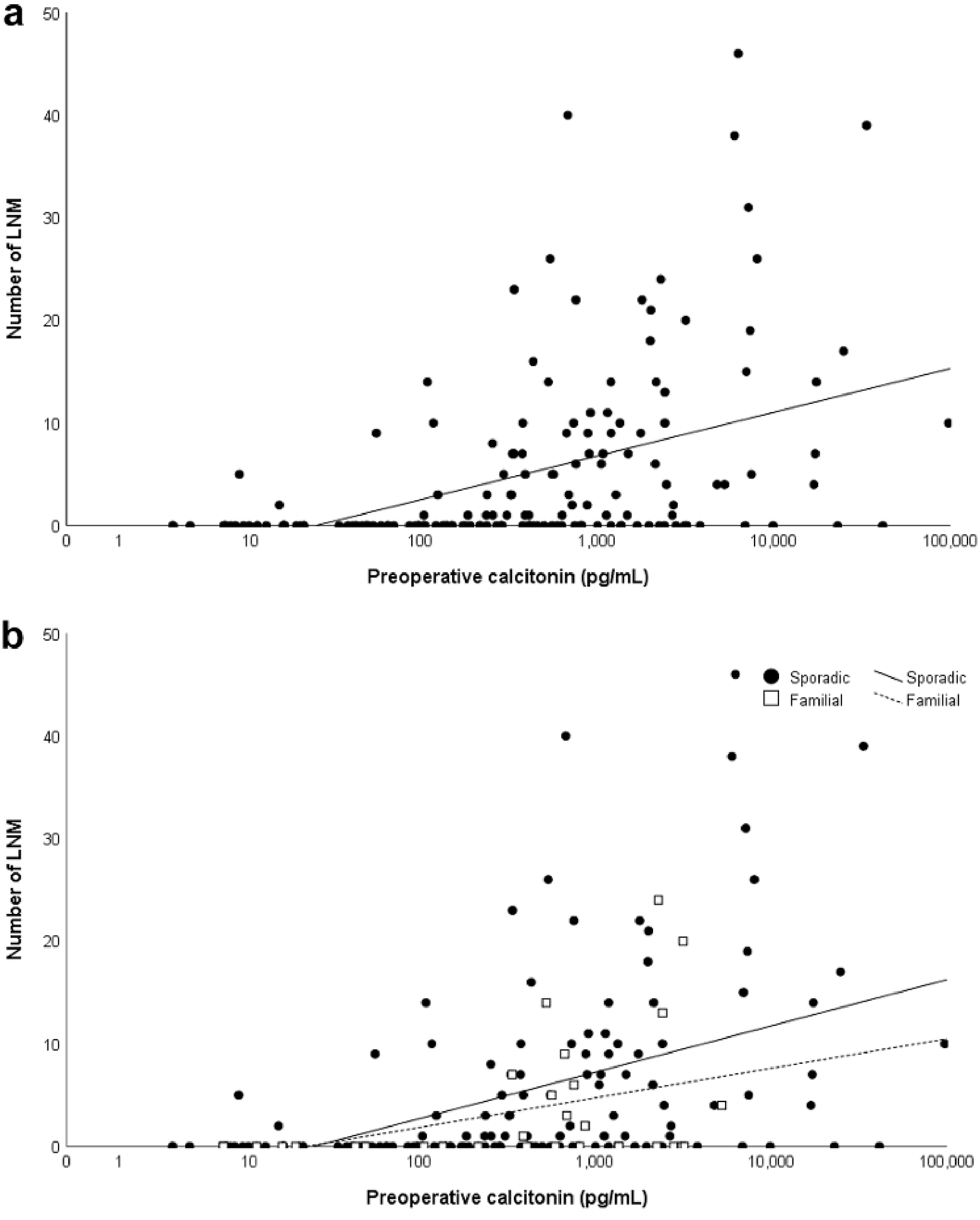


**Figure S2.** Correlation between preoperative log-calcitonin and the number of LNMs. (A) All patients regardless of tumour type ($R^2 = 0.201$ and $p < 0.001$). (B) Patients divided into sporadic and hereditary medullary carcinoma groups ($R^2 = 0.212$ and $p < 0.01$ for sporadic medullary thyroid carcinoma; $R^2 = 0.148$ and $p = 0.032$ for hereditary medullary thyroid carcinoma).

**Table S1.** Clinical course and pre- and post-operative calcitonin and images in 11 patients who did not undergo central lymph node dissection.

| No | Preop Calcitonin (pg/mL) | Primary tumor size (cm) | Multi-focality | Postop Calcitonin (pg/mL) | Preop US | Preop CT | Postop US or CT | Clinical Course |
|---|---|---|---|---|---|---|---|---|
| 1 | 3 | 0.3 | No | - | No LNM | - | - | Loss of follow-up |
| 2 | 16 | 0.3 | No | 8 | No LNM | No LNM | No LNM | No recurrence |
| 3 | 46 | 0.7 | No | 8 | No LNM | - | No LNM | No recurrence |
| 4 | 50 | 0.2 | No | 2 | No LNM | - | No LNM | No recurrence |
| 5 | 86 | 1.2 | No | 7 | No LNM | No LNM | No LNM | No recurrence |
| 6 | 98 | 0.9 | No | 72 | No LNM | - | No LNM | Recurrence in operative bed |
| 7 | 106 | 3.4 | No | 40 | No LNM | - | No LNM | No recurrence |
| 8 | 217 | 0.4 | Yes | 3 | No LNM | - | No LNM | No recurrence |
| 9 | 597 | 4.0 | No | 1 | No LNM | No LNM | No LNM | No recurrence |
| 10 | 1370 | 1.2 | Yes | 2 | - | No LNM | No LNM | No recurrence |
| 11 | 2251 | 2.0 | No | 4 | No LNM | No LNM | No LNM | No recurrence |

No = Number; Preop = pre-operative; Postop = post-operative; US = thyroid ultrasound; CT = computer tomography; LNM = lymph node metastasis.

© 2020 by the authors. Licensee MDPI, Basel, Switzerland. This article is an open access article distributed under the terms and conditions of the Creative Commons Attribution (CC BY) license (http://creativecommons.org/licenses/by/4.0/).
